# Role of *Drosophila* in Human Disease Research 2.0

**DOI:** 10.3390/ijms23084203

**Published:** 2022-04-11

**Authors:** Masamitsu Yamaguchi, Shinya Yamamoto

**Affiliations:** 1 Kansai Gakken Laboratory, Kankyo Eisei Yakuhin Co., Ltd., 3-6-2 Hikaridai, Seika-cho, Kyoto 619-0237, Japan; 2 Department of Molecular and Human Genetics, Baylor College of Medicine, Houston, TX 77030, USA; yamamoto@bcm.edu; 3 Jan and Dan Duncan Neurological Research Institute, Texas Children’s Hospital, Houston, TX 77030, USA

The fruit fly *Drosophila melanogaster* is a highly tractable animal model to study various human diseases [1]. Many biological pathways are highly conserved between humans and flies, and nearly 85% of human disease-causing genes have homologs in *Drosophila*. *Drosophila* has a relatively short life span and produces a large number of progenies, allowing researchers to perform rapid experiments that can easily be reproduced, allowing reliable statistical analyses of biological data. In addition, there are fewer ethical concerns when using *Drosophila* for biomedical research compared to mammalian models. Importantly, sophisticated genetic experiments can be designed to answer complicated biological questions, and many mutants and transgenic lines are readily available from the public stock centers. As a result, this model system is now being actively used not only to studying in vivo functions of human disease-related genes, but also to screen and evaluate candidate substances for therapeutic research.

The first volume of this special edition included 11 manuscripts (https://www.mdpi.com/journal/ijms/special_issues/Drosophila_Human_Diseases, accessed on 5 April 2022) which showcased specific examples or gave a general overview of the value of using *Drosophila* models to study a wide variety of human diseases. The diseases covered in the first volume include Alzheimer’s disease, epileptic encephalopathy, autism spectrum disorders, *phosphoribosyl pyrophosphate synthetase* (*PRPS*)-associated disorders, the transcription and nucleotide excision repair factor *TFIIH*-related diseases, central nervous system disorders associated with glial defects, *multi sex combs* (*mxc*)-associated lymphoma and alcohol use disorder. The second volume of this special edition (https://www.mdpi.com/journal/ijms/special_issues/Drosophila_Human_Diseases_2, accessed on 5 April 2022) further expands this list to cover other human diseases and disorders, such as oral cancer in relation to noncoding microRNA (miRNA), obesity, maturity-onset diabetes of the young type 2 (MODY-2), Parkinson’s disease (PD), amyotrophic lateral sclerosis (ALS), Charcot-Marie-Tooth disease (CMT) and infectious diseases through eight additional articles as summarized below.

Three research articles published in this volume showcase that *Drosophila* biologists can contribute to mechanistic and therapeutic studies of both common and rare diseases. In the first study, Jung et al. took a hybrid approach combining in vivo experiments in *Drosophila* with in vitro experiments using oral squamous cell carcinoma (OSCC) human cell lines to study the roles of miRNAs in pathophysiology of oral cancer. They found that both *Drosophila* and human miRNA-31 (miR-31) regulates expression of *wntless*, a regulator of Wnt signaling pathway that has been linked to tumorigenesis and progression of various tumors [2]. This hybrid approach can be further extended to studying the role of specific miRNAs and various human cancers but also to understand other types of noncoding RNAs in other types of diseases. For example, *Drosophila* models can be utilized to identify and characterize the functions of specific long noncoding RNAs (lncRNAs) related to neurological disorders [3]. In the second study, Lee et al. utilized a high-fat diet induced *Drosophila* obesity model to study the effect of Gomisin N (GN), a lignin derived from *Schisandra chinensis* [4]. The fly model utilized for this study shows a number of parallels to obese human, exhibiting increased body mass, decreased locomotive ability and shortened life span. Administration of GN suppressed the obesity-related phenotypes in *Drosophila*, suggesting GN as a potential agent to prevent or treat obesity. In the third study, Mascolo et al. generated a fly model of MODY-2, a rare human disorder caused by pathogenic variants in *glucokinase* (*GCK*). By knocking down the fly homologs of *GCK*, the authors found that this manipulation leads to genomic instability, likely caused by reactive oxygen species production triggered by advanced glycation end-products [5]. The authors further showed that treating MODY-2 model flies with an antioxidant vitamin B6 can suppress this phenotype, suggesting potential therapeutic avenues for this disorder.

Three of the five review articles published in this volume highlights seminal contributions made by *Drosophila* biologists that significantly deepened our mechanistic understanding of various neurological disorders. PD is a common neurodegenerative disorder that is characterized by the progressive degeneration of dopaminergic neurons. α-Synuclein (αSyn) plays a major role in the pathogenesis of PD. Suzuki et al. summarized novel insights obtained through recent studies using various *Drosophila* PD models overexpressing human αSyn [6]. The authors discuss that various characteristics of PD are recapitulated in these *Drosophila* PD models and that majority of PD likely result from complex interaction of multiple genetic factors and αSyn. αSyn-expressing *Drosophila* PD models have been particularly useful in studying the function of a number of risk genes that have been identified in PD and their relationship with αSyn. Further characterization of these genes would be important to better understand the molecular pathophysiology of PD.

CMT is a type of inherited peripheral neuropathy, displaying slow progressing muscle weakness and sensory loss in a distal dominant pattern. More than 100 genes related to CMT have been identified so far. Kitani-Morii and Noto summarized recent insights obtained from studying *Drosophila* CMT models, covering molecules associated with mitochondria, endosomes/lysosomes, tRNA, axonal transport, and glucose metabolism [7]. Taking advantage of the fact that motor symptoms of CMT are recapitulated in many *Drosophila* CMT models, genetic interaction studies performed using these models are now advancing CMT research. Especially, *Drosophila* studies are beginning to provide molecular insights into phenotypic heterogeneity in CMT, elucidating potential genes and mechanisms that alter the characteristics of the disease presentation such as affected sites and age of onset, progression rate, and duration of disease. Similar phenotypic heterogeneity is also observed in patients with ALS, a progressive and devasting neurodegenerative disease. Identification of cellular mechanisms underlying heterogeneity of these and other disorders is important, since they could provide potential targets for the development of preventive or therapeutic strategies. Epigenetic regulation and its disruption may be one of such mechanisms that serve as important factors, dictating the initiation and/or progression in CMT and ALS. Yamaguchi et al. summarized pioneering studies using *Drosophila* models that linked ALS and CMT to epigenetic regulation associated with post-translational histone modifications and noncoding RNAs [8]. Whole-exome sequencing analyses are becoming more commonly used for the genetic diagnosis of ALS and CMT. However, findings in *Drosophila* models reveal the importance of obtaining information from noncoding regions of the genome, recommending patients and clinicians to gain access to other types of genetic testing tools such as whole-genome sequencing for deeper understanding of the pathophysiology of both these disorders. Indeed, the authors discuss that genetic approaches using *Drosophila* models identified several epigenetic regulators as important modifiers of phenotypes seen in ALS and CMT model flies. Further characterization of these factors in relation to these diseases would be an important future direction, not only for ALS and CMT research but also for studies related to other types of neurological and neurodegenerative disorders.

The recent COVID-19 pandemic has increased the importance of studying infectious diseases. In a comprehensive review article published in this volume, Harnish et al. introduced studies that used transgenic *Drosophila* strains to overexpress pathogenic proteins produced by viruses and bacteria to elucidate host-pathogen interactions in vivo [9]. Since the innate immune system, developmental signaling pathways, and many other fundamental biological pathways are conserved between humans and *Drosophila*, insights into how pathogenic proteins alter these pathways have been investigated in vivo using *Drosophila* models, especially over the past decade. Notably, these studies often result in unexpected findings, revealing that certain pathogenic proteins alters biological pathways that were initially thought be unrelated. Furthermore, the authors discuss how expression of a single or multiple pathogenic protein(s) in *Drosophila* has allowed researchers to determine which host proteins mediate the effect of certain pathogenic factors, providing useful information to shed light onto disease mechanisms. Furthermore, expression of variant forms of pathogenic proteins allows quick and efficient evaluation of evolving pathogens such as the Zika virus and SARS-CoV-2. In addition, *Drosophila* models for infectious diseases can be used to screen and evaluate the efficacy of small compounds for therapy of infectious diseases.

In the final review article in this volume, Pitchakarn et al. discuss the utility of flies as a ‘living test tube’ to rapidly screen for potential mutagens and carcinogens. In *Drosophila*, a sensitive in vivo genotoxicity test called the somatic mutation and recombination test (SMART) or wing spot test has been developed. In this article, the authors describe the principle of the SMART assay and summarize its application to various health-related industries [10]. The SMART assay evaluates the genetic damage induced in dividing wing imaginal disc cells. Such damage results in loss of heterozygosity of a visible marker during larval development, which can easily be visualized as mutant wing spots in adult flies. Since the SMART assay is high-throughput, economic and can be used to assess food safety, drug safety and genotoxicity of various environmental pollutants, the authors argue that various health-related industries can benefit from this technology. This review also provides a glimpse to the recent trend that various industrial applications of *Drosophila* are also being actively explored and adopted. For instance, many companies all over the world now provide outsourcing services in generating transgenic flies and gene knockout/knockin strains by offering microinjection and genome editing services in *Drosophila*. Some companies perform screening and evaluation of candidate drugs and bioactive substances using wild-type or various human disease *Drosophila* models. Cooperations and collaborations that cross-pollinate scientific community, medical society and industry provide exciting opportunities to greatly advance biomedical sciences [11].

In summary, the role of *Drosophila* in human disease research has been exponentially increasing, especially over the past two decades. In addition to elucidating fundamental mechanisms of conserved biological pathways involved in both rare and common diseases, *Drosophila* researchers are making breakthroughs in translational and clinical research. Excitingly, this is happening not only in academia but also in biotech and pharmaceutical industries. We hope the 19 articles featured in the first and second volume of our special edition of *International Journal of Molecular Sciences* provide the readership of this journal with exciting examples of *Drosophila* research that are making major contributions to human disease research. We look forward to the third volume of this special edition, which will likely reveal and highlight additional medical disciplines that benefit from utilization of *Drosophila* in research and development.
